# Effect of Rice Protein on the Gelatinization and Retrogradation of Rice Starch with Different Moisture Content

**DOI:** 10.3390/foods13233734

**Published:** 2024-11-22

**Authors:** Yifu Zhang, Jiawang Zhang, Zeyu Wang, Longxiang Fan, Ye Chen

**Affiliations:** College of Food Science and Engineering, Tianjin University of Science and Technology, Tianjin 300457, China

**Keywords:** rice starch, rice protein, moisture content, starch retrogradation

## Abstract

Rice protein and moisture content are pivotal in the gelatinization and retrogradation processes of rice starch. This study aimed to explore the influence of rice protein on these processes by preparing rice starch gels with varying moisture levels and incorporating rice protein. At a high moisture content of 1:6, rice protein exhibited a minimal effect on the gelatinization properties of rice starch but significantly retarded the retrogradation of the starch gel. At intermediate moisture levels of 1:4 and 1:2, the rice starch gels showed pronounced retrogradation. However, rice protein was effective in inhibiting this retrogradation at a 1:4 moisture content, while its inhibitory effect diminished at a 1:2 moisture content. Under low moisture conditions of 1:1, the gelatinization of rice starch was markedly constrained by the limited water availability, but rice protein mitigated this constraint. Conversely, at this moisture level, rice protein promoted the retrogradation of the rice starch gel during the retrogradation process. The findings of this study offer a theoretical foundation that could inform the production of rice-based products.

## 1. Introduction

Rice is one of the most important crops in the world, a major food crop in many countries around the world, and a basic food source for human beings [1,2]. Starch is an important component of rice and rice processed foods [3]. Starch gelatinization and retrogradation play an important role in determining the quality of rice products [4]. The gelatinization of starch has an impact on both the eating and cooking quality of rice [5]. During the retrogradation of starch, transportation and storage not only affect the sensory quality of starch products, but also greatly reduce their nutritional value [6,7]. After starch retrogradation, it forms resistant starch, which is difficult for the gastrointestinal tract to digest and absorb. Excessive consumption may lead to indigestion, bloating, and other symptoms. For individuals with weaker gastrointestinal function, consuming too much resistant starch may burden the gastrointestinal system, causing discomfort [8]. Additionally, the gel structure formed by retrograded starch can encapsulate other nutrients, such as proteins and fats. This encapsulation makes these nutrients less accessible to digestive enzymes, further reducing the nutritional bioavailability of the food [9]. Therefore, by regulating the gelatinization and retrogradation of rice starch, it is possible to improve the viscosity and texture of rice products, enhance their water retention, and increase their eating quality and shelf life during storage. This is crucial for enhancing the stability of food products and holds significant reference value for processing techniques in the rice food industry.

The interaction between proteins and starch has a profound effect on the gelatinization and retrogradation properties of rice and rice products [10,11]. Wu et al. showed that the viscosity and enthalpy of gelatinization of rice starch decreased gradually with the increase of rice protein, while the gelatinization temperature increased gradually, indicating delayed gelatinization of rice starch [12]. The study by Baxter et al. mentioned that the addition of 1–10% rice glutenin to rice starch decreased its peak and final viscosities, while increasing the gelatinization temperature in a concentration-dependent manner [13]. The interaction of amino acids with starch increased the ordered and aggregated structure of rice starch [14]. Moisture is essential in the processing of rice products and moisture content also affects the gelatinization and retrogradation of rice starch [15,16]. As the moisture content increased, the gelatinization transmittance, gelatinization temperature, and digestibility of starch increased, while the swelling power and enthalpy decreased [17]. As the degree of starch gelatinization increased with increasing moisture content, the number of disordered amylose and amylopectin molecules available for nucleation increased, and the rate of migration of amylose and amylopectin increased, resulting in the formation of more crystals [18]. Higher moisture content favored the recrystallization of amylopectin, while moisture content had little effect on the recrystallization of amylose [19]. However, the quality changes during the processing and storage of rice products are the result of the interaction of multiple factors, including starch, protein, water, and others. If we only study the impact of a single component on the gelatinization and retrogradation properties of rice starch, it is far from sufficient. Therefore, investigating the interaction of multiple factors can provide a more accurate understanding of the actual changes that occur during the processing and storage of products. The effect of protein–starch interaction on the gelatinization and retrogradation of rice starch may change at different moisture contents. In summary, existing research has primarily focused on the impact of single components on the gelatinization and retrogradation of rice starch. However, the processing of rice starch products involves the interplay of various substances, including rice protein and moisture. Therefore, it is essential to investigate the interactions of the ternary system comprising rice protein, moisture, and rice starch during starch gelatinization and retrogradation. This research is crucial for enhancing the processing, storage quality, and digestibility of rice starch products.

Thus, this study investigated the differences in interactions between proteins and starch under different water conditions. Clarified the different effects of rice protein on the gelatinization and retrogradation properties of rice starch under different moisture conditions. According to our previous research [20] and related research results [12], rice protein had a dose-dependent effect on the gelatinization and retrogradation of rice starch. With the increase in protein content, rice protein hindered the gelatinization of rice starch and had an inhibitory effect on the retrogradation of rice starch. At a rice protein content of 10%, the protein exhibited the most pronounced effect in inhibiting the retrogradation of rice starch. However, as the protein content exceeded 10%, this inhibitory effect began to significantly diminish. Therefore, rice starch gel with 10% rice protein was selected as the experimental group and pure rice starch gel as a control group to explore the difference in the effect of rice protein on the gelatinization and retrogradation properties of rice starch under different moisture content. This study obtained the mechanism of action of rice protein and moisture together on rice starch gelatinization and retrogradation, which was important for improving the quality and taste of rice products. The research results provide a reference for the study of the interaction mechanisms of substances within rice itself and lay a theoretical foundation for optimizing the processing technology of rice products to enhance product quality.

## 2. Materials and Methods

### 2.1. Materials

Rice starch (Product Number: S7260, PubChem CID: 24836924) was purchased from Sigma Aldrich Co., LLC. (Shanghai, China). Fulinmen Akita Komachi rice (Product Code: 6944910328431) was purchased from China Cereals, Oils and Foodstuffs Group Co. (Beijing, China). Sodium hydroxide (NaOH, Analytical Reagent) was purchased from Jindong Tianzheng Fine Chemical Reagent Factory (Tianjin, China). Hydrochloric acid (HCl, Analytical Reagent) was purchased from Tianjin Chemical Reagent Supply and Marketing Company (Tianjin, China).

### 2.2. Extraction of Rice Protein

Rice was pulverized and sieved by a 100-mesh sieve using an electric powderer (LK-1000A, Zhejiang Wenling Chuangli Medicinal Machinery Factory, LLC., Taizhou, China), dissolved the rice flour in 0.3% NaOH (PubChem CID: 14798) solution in the ratio of 1:6, and extracted at room temperature for 4 h with occasional stirring during the extraction period. Extracts were centrifuged at 4500× *g* for 10 min. The pH of the supernatant was adjusted to 6.3 by concentrated hydrochloric acid (PubChem CID: 313), 4500× *g* centrifuged for 10 min. The precipitate was washed repeatedly with distilled water until neutral. Precipitates were dispersed in distilled water and put into a dialysis bag (3500 D) to remove soluble salts, freeze-dried, and ground. The content of rice protein was determined to be 90.74% ± 0.29 (dry basis) using a BCA protein assay kit (Beijing Solarbio Science & Technology Co., Ltd., Beijing, China).

### 2.3. Differential Scanning Calorimetry (DSC)

Accurately weigh 1 mg of rice starch in an aluminum crucible, added 6 μL, 4 μL, 2 μL, and 1 μL of distilled water to the crucible according to the ratios of 1:6, 1:4, 1:2, and 1:1, respectively. The lid was pressed tightly and oscillated evenly. After equilibrium at room temperature for 2 h, the DSC (DSC-60A, SHIMADZU Co., Ltd., Kyoto, Japan) tester was used for determination. The test temperature was raised from 30 °C to 100 °C of 10 °C per minute for heating rate. After the test, the analysis was carried out by the computer‘s own software.

The rice protein sample was mixed with rice starch in the mass ratio of 10:90 to obtain a sample containing 10% rice protein for testing. Accurately weigh 1 mg of the sample to be tested in an aluminum crucible, added 6 μL, 4 μL, 2 μL, and 1 μL of distilled water to the crucible according to the ratios of 1:6, 1:4, 1:2, and 1:1, respectively. The lid was pressed tightly and oscillated evenly. After equilibrium at room temperature for 2 h, the DSC tester was used for determination. The test temperature was raised from 30 °C to 100 °C of 10 °C per minute for heating rate. After the test, the analysis was carried out by the computer‘s own software.

### 2.4. Dynamic Rheometer

Accurately weighed 2 g of rice starch in a beaker, according to the ratio of 1:6, 1:4, 1:2, and 1:1, to the beaker were added 12 mL, 8 mL, 4 mL, and 2 mL of distilled water, stirring uniformly to produce different concentrations of starch to be tested solution. Dynamic rheometer (HAAKE MARS 60, Thermo HAAKE., Ltd., Karlsruhe, Germany) was used for the determination, the test rotor was selected as a flat rotor with a diameter of 20 mm, and 1 mL of starch to be tested was sucked up and dripped on the test plate. The test program was a continuous scanning of oscillating temperature. Test temperature was firstly warmed up from 30 °C to 95 °C at a rate of 5 °C per minute, lowered down to 30 °C from 95 °C with the same rate, with a strain of 1.0%, and 200 data points were taken for each of the heating and cooling.

The rice protein sample was mixed with rice starch in the mass ratio of 10:90 to obtain a sample containing 10% rice protein for testing. Accurately weighed 2 g of the sample to be tested in the beaker, according to the ratio of 1:6, 1:4, 1:2, and 1:1, added 12 mL, 8 mL, 4 mL, and 2 mL distilled water to the beaker. Stirred evenly to prepare different concentrations of the test solution. A dynamic rheometer was used for the determination, the test rotor was selected as a flat rotor with a diameter of 20 mm, and 1 mL of the liquid to be tested was sucked up and dropped on the test plate. The test program was a continuous scanning of oscillating temperature. Test temperature was firstly warmed up from 30 °C to 95 °C at a rate of 5 °C per minute, lowered down to 30 °C from 95 °C with the same rate, with a strain of 1.0%, and 200 data points were taken for each of the heating and cooling.

### 2.5. Sample Preparation for the Retrogradation of Rice Protein, Moisture, and Rice Starch Mixtures

The rice protein sample was mixed with rice starch at a mass ratio of 10:90 to obtain a sample containing 10% rice protein. The sample was weighed into a centrifuge tube, and distilled water was added in the ratios of 1:6, 1:4, 1:2, and 1:1, respectively, and shaken to disperse the sample evenly. The tubes were heated in a boiling water bath for 20 min with occasional shaking. After heating, the centrifuge tubes were cooled to room temperature and then stored in a refrigerator at 4 °C for 0, 1, 3, 7, 14, and 21 days. The samples were taken out of the refrigerator for further characterization after different days of storage.

### 2.6. Low-Field Nuclear Magnetic Resonance (LF-NMR)

After the starch gel samples stored for different days were removed from the refrigerator, the centrifuge tubes were equilibrated in a water bath at 32 °C for 10 min, and then the centrifuge tubes were placed into 25 mm NMR tubes to determine the transverse relaxation time by using LF-NMR analyzer (MicroMR-25, Niumag Co., Ltd., Suzhou, China). The Carr–Purcell–Meiboom–Gill (CPMG) sequence was selected for 0.35 ms echo time, 6000 echoes, and 4 scanning times. The resulting inversion images were analyzed by the software.

### 2.7. Low-Field Magnetic Resonance Imaging (LF-MRI)

The starch gel samples stored for different days were removed from the refrigerator and the centrifuge tubes were equilibrated in a water bath at 32 °C for 10 min. Subsequently, the centrifuge tubes were placed into 25 mm MRI tubes, and the imaging measurements were carried out using the LF-MRI system (MicroMR-25, Niumag Co., Ltd., Suzhou, China). The imaging test was selected as an SE sequence of 100 mm × 100 mm field of view, 20 ms echo time, 500 ms recovery time, and 4 scanning times. The obtained images were processed with the pseudo-color software for color processing.

### 2.8. Scanning Electron Microscope (SEM)

Starch gel samples stored for different days were removed from the refrigerator, and the samples were spread in a glass dish with a thickness of about 0.5 mm, pre-frozen at −40 °C, and freeze-dried. Samples were quenched using liquid nitrogen, and the cross-sectioned samples were affixed to a fluted plate with double-sided adhesive tape, and then observed with SEM (SU1510, Hitachi Science Systems, Ltd., Tokyo, Japan) after gold spraying with an ion sputterer. The operating voltage was 1 kV and the magnification was 100 times.

### 2.9. Statistical Analysis

All data statistics were analyzed using SPSS 22.0 software (22.0, SPSS Institute, New York, NY, USA). The average (AVG) and standard deviation (SD) were calculated by repeating the experiment three times. Significance was analyzed using the Tukey (John Wilder Tukey) model with a significant level of 0.05. Images were drawn using OriginPro 2016 software.

## 3. Results and Discussion

### 3.1. Thermodynamic Properties of Rice Starch with Rice Protein and Moisture

The changes in the onset temperature (T_o_), peak temperature (T_p_), termination temperature (T_e_), and enthalpy (ΔH) during the gelatinization of rice starch without and with 10% rice protein at different moisture contents are shown in Table 1.

The data in Table 1 showed that as the moisture content decreased, both in the T_o_ and T_p_ of gelatinization of rice starch did not change significantly, which were related to the type and structure of the substance itself. This indicated that changes in moisture content and the combined effect of rice protein and moisture did not cause alterations in the structural properties of rice starch during the heating process. The gelatinization ΔH also showed no significant difference, suggesting that all samples reached the same degree of gelatinization after the same DSC heating program [21]. T_e_ indicated the temperature at which the gelatinization process is completely completed. For rice starch without rice protein, the T_e_ of the gelatinization showed a significant increase under conditions of high moisture content (1:6), medium moisture content (1:2), and low moisture content (1:1). This was because the gelatinization of starch required the participation of water. As the moisture content decreased, water gradually became the main limiting factor for starch gelatinization. The water absorption and swelling of starch granules were constrained, leading to an increase in the T_e_ [22,23]. However, for rice starch with 10% rice protein, the T_e_ of the gelatinization only showed a significant increase under the condition of low moisture content (1:1). This indicated that the addition of rice protein could, to some extent, reduce the dependence of the rice starch gelatinization on water [24].

### 3.2. Rheological Properties of Rice Starch with Rice Protein and Moisture

**Figure 1:**
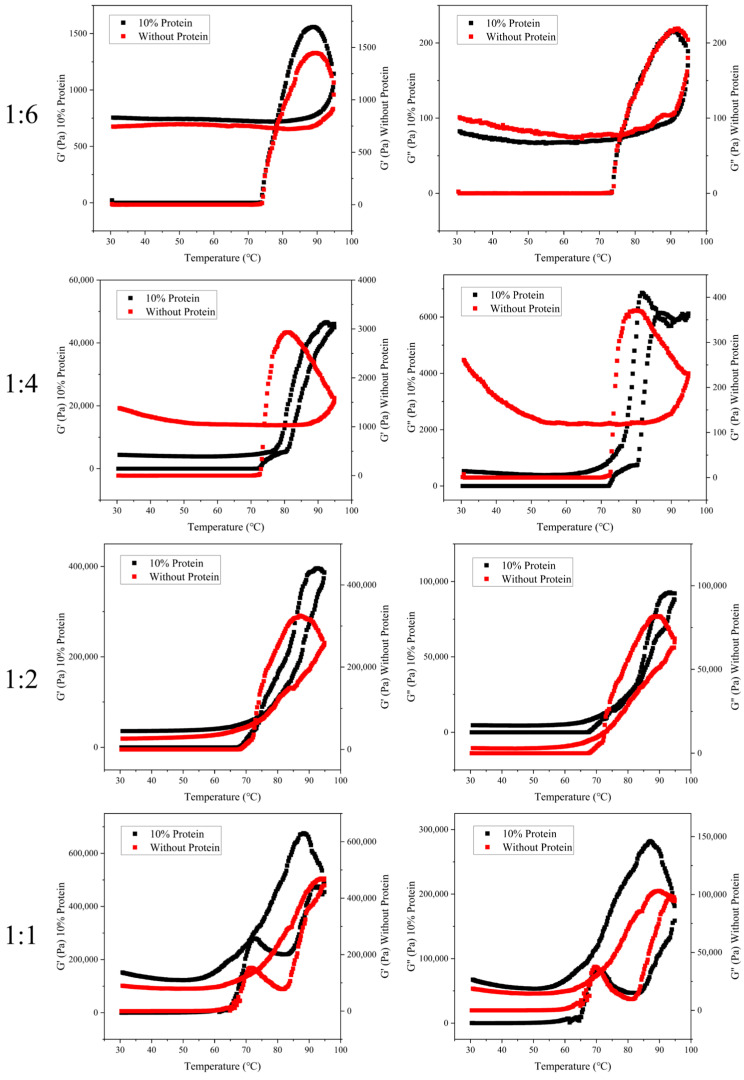
The G′ and G″ of rice starch without and with 10% rice protein during heating at different moisture contents.

The changes in storage modulus (G′) and loss modulus (G″) of rice starch without and with 10% rice protein at different moisture contents during heating were shown in Figure 1.

With the continuous reduction of moisture content, the rheological properties of rice starch changed, and only under the condition of low moisture content (1:1), the G′ and G″ of rice starch change greatly during the heating process, and at this time, the changes in the heating process can be divided into four stages. In the first stage, the G′ and G″ increased due to the continuous water absorption and swelling of rice starch granules in the early stage of heating; in the second stage, due to the larger concentration of starch, the overall viscosity of the system was higher, which led to the melting of the crystal structure as well as the separation of the amylose and amylopectin, which was clearly manifested in the reduction of G′ and G″; in the third stage, due to the dissolution and cross-linking of amylose and amylopectin, a network structure was formed, which led to a rapid increase in the G′ and G″; in the fourth stage, the starch network-like structure was disintegrated due to continuous high-temperature heating, which led to a decrease in the G′ and G″ [25,26]. At higher moisture contents, the second stage was not significantly detected due to the low viscosity of the whole system. At high moisture content (1:6), the thickening effect of rice protein was not obvious, but as the moisture content decreased, the thickening effect of rice protein was significantly manifested and the viscosity of the whole system increased. The addition of rice protein led to an increase in the G′ and G″ of starch at the same moisture content, indicating that the gel strength of the starch gels obtained with rice protein was greater [27].

During the cooling process, starch underwent retrogradation, causing the starch gel to become harder and stronger. This results in an increase in the G′ and G″ towards the end of the cooling process [28]. As shown in Figure 1, under the condition of medium moisture content (1:4), there was a significant increase in the G′ and G″ at the end of the cooling process, indicating that significant retrogradation of rice starch gel occurs at this stage. This phenomenon suggested that as the water content decreased, the rate of retrogradation of rice starch first increased and then decreased. At higher water contents, as the water content decreased, the probability of collisions between starch molecules increased, making it easier for cross-linking to occur, leading to noticeable retrogradation. However, at lower water contents, the movement of starch molecules was restricted, which hindered the cross-linking of starch molecules. Additionally, the lack of water participation results in less pronounced retrogradation. When rice protein was added, under the condition of medium water content (1:4), there was no significant increase in the G′ and G″ at the end of the cooling process, indicating that the retrogradation of starch was no longer prominent. This was because the addition of rice protein restricted the collisions between amylose molecules in rice starch, inhibiting the cross-linking between molecules and thus suppressing the retrogradation of starch. However, under the condition of low water content (1:1), the retrogradation of starch was more pronounced compared to pure starch at the same water content, suggesting that the addition of rice protein promotes the retrogradation of rice starch. This was due to the hygroscopic nature of rice protein, which caused water to migrate from the rice protein to the rice starch when the starch was forming an ordered structure and experiencing water loss. This migration compensated for the water deficiency, ensuring the formation of an ordered structure.

### 3.3. Rice Protein and Moisture on the Microstructure of Rice Starch During Retrogradation

The data regarding the property changes of rice starch gels without rice protein during the retrogradation at different moisture contents were published in STARCH-STARKE [29]. In subsequent analyses, we compared the property changes of rice starch without and with 10% rice protein during the retrogradation at different moisture contents.

**Figure 2:**
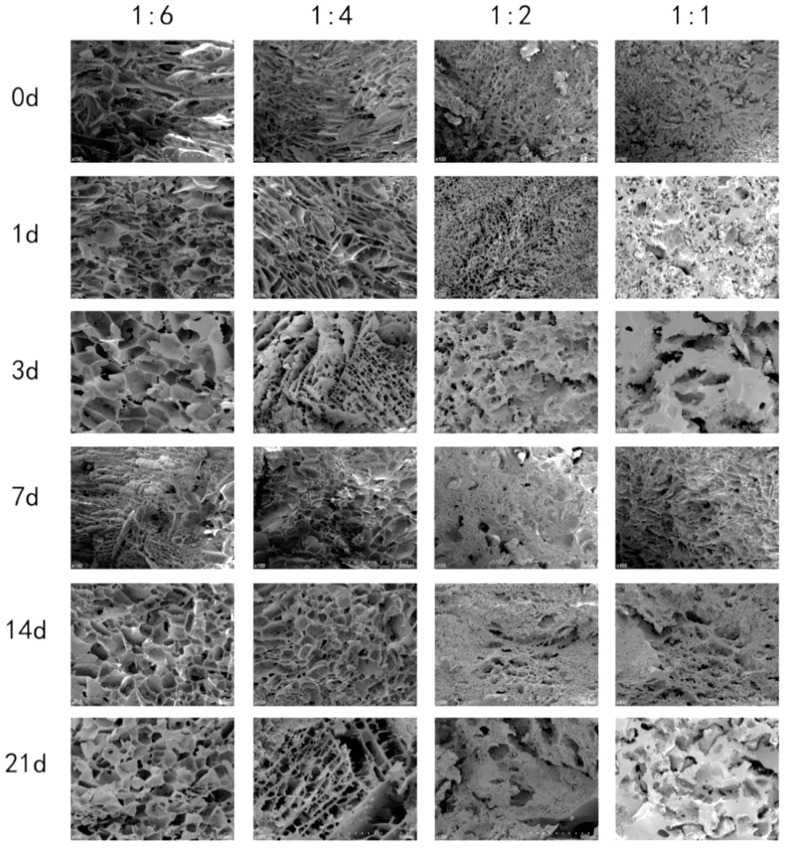
SEM images of rice starch gel samples with different moisture content and 10% rice protein on different retrograding days (×100).

To investigate the impact of the combined effects of rice protein and moisture on the microstructure of rice starch during the retrogradation, we observed the microstructure of starch gels with 10% rice protein for different retrograding days under different moisture content, as shown in Figure 2.

**Figure 3:**
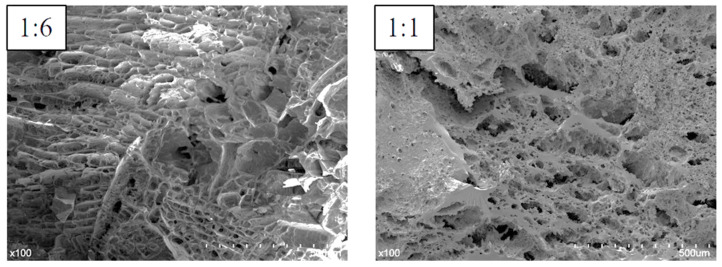
SEM images of rice starch gel samples with 10% rice protein at 1:6 and 1:1 (×100).

In Figure 2, all rice starch gel samples with rice protein exhibited a network-like structure. As the water content decreased, the walls of the network structure gradually thickened. With an increase in retrograding days, the network structure of rice starch gels without rice protein showed non-uniform pore sizes, indicating the occurrence of retrogradation. However, the network structure of rice starch gels with rice protein did not exhibit significant changes in pore size, suggesting that rice protein inhibited the retrogradation of rice starch to some extent [20,29]. The influence of rice protein on the microstructure of rice starch gels varied under different moisture content conditions, as shown in Figure 3. At a high moisture content of 1:6, small protrusions could be observed on the surface of the network structure walls of rice starch gels, while the cross-section of the walls showed no significant changes. This indicated that at higher moisture content, rice protein was dispersed and attached to the surface of rice starch [30]. At a low moisture content of 1:1, more noticeable attachments were observed on the walls of the network structure of rice starch gels, and more small pores appeared on the wall structure. This suggested that, in addition to being attached to the surface of rice starch, rice protein also participated in the formation of the wall structure of rice starch gels.

### 3.4. Rice Protein and Moisture on the Transverse Relaxation Time of Rice Starch During Retrogradation

The inverse results of the multi-exponential modeling of the transverse relaxation time of rice starch gels with different moisture content and 10% rice protein at different retrograding days [31] are shown in Table 2.

As shown in Table 2, for rice starch gels with 10% rice protein at sample-to-water ratios of 1:6, 1:4, and 1:2, the transverse relaxation time of the rice starch gels exhibited three peaks, corresponding to ordered structure constituting water (T_21_), intermolecularly bound water (T_22_), and free water (T_23_) [29]. However, at a sample-to-water ratio of 1:1, the transverse relaxation time of the rice starch gels showed only two peaks. Due to the decrease in moisture content, the T-values shifted to the left, and the ordered structure constituting water and intermolecularly bound water merged into T_21_, while T_22_ was the peak of free water.

In Table 2, as the moisture content decreased, the peak values for free water and intermolecular bound water decreased, while the peak value for ordered structure constituting water did not change significantly. This was because, with the decrease in moisture content, the pores in the starch gel network structure became smaller, leading to stronger binding of free water within the pores and reduced mobility. As the wall of the starch network structure continued to thicken, a large number of starch molecules were aggregated, which made the degree of intermolecular bound water greater, thus leading to a decrease in mobility. The ordered structure constituted water because it was inside the ordered structure formed by starch retrogradation, so the mobility change was not obvious.

Comparing starch gel samples aged for different retrograding days, the peak value of free water in rice starch gels without rice protein showed a stepwise decrease with increasing retrograding days, and the peak value of intermolecular bound water also showed a gradually decreasing trend [29]. However, in rice starch gels with rice protein, the peak value of intermolecular bound water did not change significantly. This was because, at higher moisture content, rice protein attached to the surface of rice starch molecules. During the retrograding, rice protein restricted the collision of starch molecules, delayed the formation of ordered structures, and inhibited the retrogradation of rice starch gels.

### 3.5. Rice Protein and Moisture on Moisture Distribution of Rice Starch During Retrogradation

**Figure 4:**
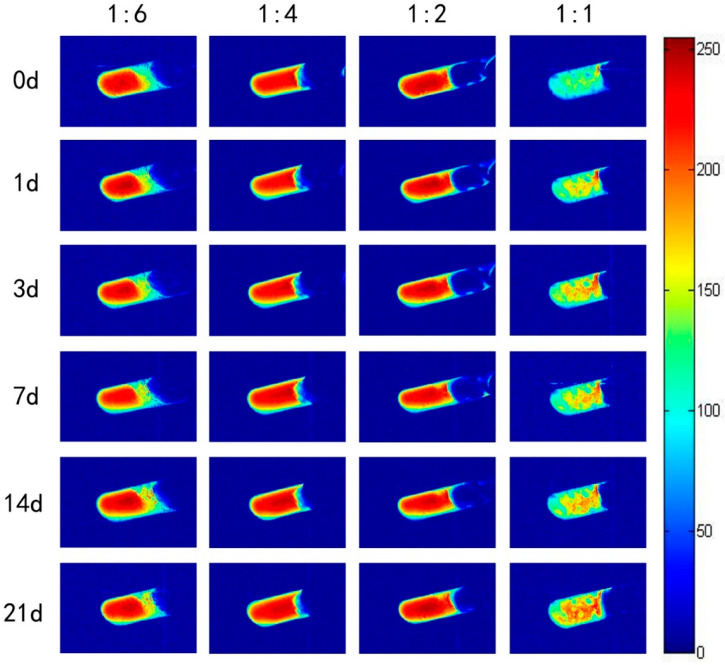
NMR images of rice starch gel samples with different moisture content and 10% rice protein on different retrograding days.

In order to further investigate the effect of the addition of rice protein at different moisture content on the moisture distribution of rice starch gels at different retrograding days, the changes in the proportion of the peak areas of the peaks of the transverse relaxation times of the rice starch gels with different moisture content and the addition of 10% of rice protein at different retrogradation days were presented in Table 3, and the corresponding LF-NMR images were shown in Figure 4.

From the data in Table 3, it could be observed that as the number of retrograding days increased, the proportion of intermolecularly bound water of starch decreased, while the proportion of free water increased. The proportion of ordered structures constituting water remained relatively unchanged. This indicated that during the retrogradation, starch molecules cross-link with the participation of intermolecularly bound water of starch to form an ordered structure. This process converted some of the intermolecularly bound water of starch into an ordered structure constituting water. After the formation of the ordered structure, the starch molecules’ ability to bind with water decreased, causing the remaining water to be expelled and converted into free water. Therefore, the proportion of intermolecularly bound water of starch decreased, while the proportion of free water increased. During the process of stacking and extending the ordered structure, some water was expelled, resulting in the proportion of ordered structure constituting water remaining relatively unchanged. Compared to rice starch gels without rice protein, rice starch gels with rice protein had a higher proportion of ordered structure constituting water and intermolecularly bound water. This was because rice protein bound a certain amount of water and participated in the molecular cross-linking and formation of the ordered structure during the starch retrogradation.

In the LF-NMR images, the red areas represented high moisture content, the blue areas represented low moisture content, and the dark blue represented the background with a moisture content of 0. The color changes reflected the water migration process in rice starch gels during the retrogradation. With the occurrence of retrogradation, significant moisture migration occurred within the rice starch gel, leading to a decrease in the water-holding capacity of the gel, water loss, and volume shrinkage of the gel. From Figure 4, it could be observed that at higher moisture contents, the addition of rice protein, due to its good water absorption and thickening properties, enhanced the overall water-holding capacity of the rice starch gel, and there was no significant phenomenon of gel water loss and shrinkage [32]. However, when the water content was low (1:1), the high viscosity of the sample caused it to separate from the centrifuge tube wall during the mixing process with moisture, resulting in a large number of pores, which significantly affected the imaging results. Nevertheless, the images still showed a very clear water migration phenomenon, indicating significant retrogradation of the rice starch gel. Combined with the analysis of various moisture proportions in the LF-NMR measurements, this might be because at low moisture contents, the water content was insufficient to support the further formation of an ordered structure in the starch. In this case, the water bound by the rice protein migrated to the rice starch to compensate for the lack of water needed to form an ordered structure. Therefore, at low moisture contents, rice protein promoted the retrogradation of rice starch.

## 4. Conclusions

Under different moisture content conditions, rice protein had an effect on the gelatinization and retrogradation of rice starch. It was shown that there was a significant difference in the T_p_ of rice starch gels containing rice proteins under different moisture content conditions, indicating that the addition of rice proteins reduced the moisture dependence of the rice starch gelatinization to a certain extent. Under the condition of high moisture content 1:6, the addition of rice protein provided spatial restriction to inhibit the collision cross-linking between starch molecules, and at the same time, because of the water absorption of rice protein itself, the water-holding capacity of rice starch gel was enhanced. The addition of rice protein attenuated the moisture dependence of rice starch retrogradation and inhibited the retrogradation rate of rice starch. Under the condition of 1:2 medium moisture content, water migration and redistribution between rice protein and rice starch were produced, which reduced the limiting effect of moisture content on rice starch gelatinization to a certain extent. Under this condition, the retrogradation rate of rice starch gels with rice protein showed a decreasing trend. Under the condition of low moisture content of 1:1, attachments appeared on the wall of the network structure of rice starch gel, and more small pores appeared on the wall structure, which indicated that rice protein was involved in the formation of the wall structure of rice starch gel in addition to attaching to the surface of rice starch, and also indicated that rice protein promoted the retrogradation of rice starch gel, but because of its own better water-absorbing and thickening. However, because of the better water absorption and thickening effect of rice protein, the rice starch gel maintained a high water-holding capacity. The results of this study can provide reference and suggestions for the industrialized production of rice in the future.

## Figures and Tables

**Table 1:** The thermal properties of rice starch without and with 10% rice protein at different moisture content.

	T_o_ (°C)	T_p_ (°C)	T_e_ (°C)	△H (J/g)
Without protein				
1:6	63.09 ± 0.52 ^a^	70.10 ± 0.38 ^a^	76.67 ± 0.62 ^c^	8.88 ± 1.39 ^a^
1:4	63.03 ± 0.52 ^a^	70.25 ± 0.12 ^a^	77.74 ± 0.77 ^bc^	9.06 ± 0.86 ^a^
1:2	62.95 ± 0.05 ^a^	70.02 ± 0.24 ^a^	79.38 ± 0.49 ^b^	8.81 ± 0.33 ^a^
1:1	62.64 ± 0.01 ^a^	70.08 ± 0.05 ^a^	84.84 ± 1.58 ^a^	7.52 ± 0.27 ^a^
With 10% protein				
1:6	62.47 ± 1.01 ^a^	70.22 ± 0.67 ^a^	77.54 ± 0.88 ^b^	8.51 ± 1.52 ^a^
1:4	62.23 ± 0.60 ^a^	69.64 ± 0.24 ^a^	76.43 ± 0.45 ^b^	7.98 ± 0.56 ^a^
1:2	62.95 ± 0.15 ^a^	70.25 ± 0.31 ^a^	79.18 ± 0.43 ^b^	7.86 ± 0.71 ^a^
1:1	63.44 ± 0.43 ^a^	70.03 ± 0.35 ^a^	84.57 ± 2.00 ^a^	6.95 ± 0.24 ^a^

Note: Data in the table were expressed as mean ± standard deviation, and values in the same column with the same letters are not significantly different (*p* < 0.05).

**Table 2:** The multiple exponential models of T_2_ of rice starch gels with different moisture content and 10% rice protein in different retrograding days.

	T_2_/ms		
	T_21_	T_22_	T_23_
1:6			
0d	0.3824 ± 0.0295 ^ab^	11.0108 ± 0.4153 ^a^	223.9982 ± 2.5439 ^a^
1d	0.4554 ± 0.0337 ^a^	10.1096 ± 0.7805 ^a^	223.9982 ± 2.5439 ^a^
3d	0.3525 ± 0.0554 ^ab^	10.1096 ± 0.7805 ^a^	196.4972 ± 2.1769 ^b^
7d	0.3719 ± 0.0891 ^ab^	9.2637 ± 0.6847 ^a^	196.4972 ± 2.1769 ^b^
14d	0.3092 ± 0.0486 ^b^	9.2637 ± 0.6847 ^a^	196.4972 ± 2.1769 ^b^
21d	0.3355 ± 0.0259 ^ab^	9.6589 ± 0.3652 ^a^	196.4972 ± 2.1769 ^b^
1:4			
0d	0.3824 ± 0.0295 ^a^	8.8684 ± 0.6847 ^a^	151.2099 ± 1.0654 ^a^
1d	0.3675 ± 0.0480 ^a^	8.4731 ± 0.8374 ^a^	151.2099 ± 1.0654 ^a^
3d	0.3845 ± 0.0554 ^a^	8.1263 ± 0.6006 ^a^	132.6453 ± 2.7356 ^b^
7d	0.3504 ± 0.0259 ^a^	8.1263 ± 0.6006 ^a^	132.6453 ± 2.7356 ^b^
14d	0.4240 ± 0.1015 ^a^	8.4731 ± 0.8374 ^a^	132.6453 ± 2.7356 ^b^
21d	0.3719 ± 0.0891 ^a^	8.8684 ± 0.6847 ^a^	132.6453 ± 2.7356 ^b^
1:2			
0d	0.3355 ± 0.0259 ^a^	4.8122 ± 0.3557 ^a^	60.4454 ± 3.7712 ^a^
1d	0.3373 ± 0.0486 ^a^	4.8122 ± 0.3557 ^a^	60.4454 ± 3.7712 ^a^
3d	0.3525 ± 0.0554 ^a^	4.4015 ± 1.0779 ^a^	53.0243 ± 0.5133 ^b^
7d	0.3205 ± 0.0315 ^a^	4.0412 ± 0.3120 ^a^	53.0243 ± 0.5133 ^b^
14d	0.3504 ± 0.0259 ^a^	3.7031 ± 0.2737 ^a^	53.0243 ± 0.5133 ^b^
21d	0.3355 ± 0.0259 ^a^	3.8611 ± 1.3404 ^a^	53.0243 ± 0.5133 ^b^
1:1			
0d	1.3540 ± 0.4213 ^a^	21.1962 ± 0.5228 ^a^	——
1d	1.2431 ± 0.0960 ^ab^	21.1962 ± 0.5228 ^a^	——
3d	0.8818 ± 0.1386 ^ab^	21.1962 ± 0.5228 ^a^	——
7d	0.9192 ± 0.1202 ^ab^	21.1962 ± 0.5228 ^a^	——
14d	0.9777 ± 0.2485 ^ab^	21.1962 ± 0.5228 ^a^	——
21d	0.6118 ± 0.1927 ^b^	21.1962 ± 0.5228 ^a^	——

Note: Data in the table were expressed as mean ± standard deviation, and values in the same column with the same letters are not significantly different (*p* < 0.05).

**Table 3:** Change in the proportion of peak area of the three peaks in different retrograding days.

	A_2_/%		
	A_21_	A_22_	A_23_
1:6			
0d	4.8058 ± 0.3097 ^ab^	5.1030 ± 0.1078 ^a^	90.0912 ± 0.4084 ^b^
1d	4.4463 ± 0.4630 ^ab^	4.9499 ± 0.1344 ^ab^	90.6037 ± 0.3885 ^ab^
3d	4.8584 ± 0.2874 ^a^	4.8026 ± 0.1531 ^abc^	90.3389 ± 0.1365 ^b^
7d	3.8560 ± 0.5008 ^b^	4.7487 ± 0.0237 ^bc^	91.3953 ± 0.4960 ^a^
14d	4.7843 ± 0.1313 ^ab^	4.7075 ± 0.0930 ^bc^	90.5083 ± 0.2185 ^ab^
21d	5.3737 ± 0.3638 ^a^	4.5310 ± 0.1191 ^c^	90.0953 ± 0.3229 ^b^
1:4			
0d	5.2930 ± 0.7888 ^a^	6.4974 ± 0.1934 ^a^	88.2096 ± 0.6276 ^a^
1d	5.6067 ± 0.9439 ^a^	6.2040 ± 0.0740 ^ab^	88.1893 ± 0.9888 ^a^
3d	5.3605 ± 0.7136 ^a^	6.0597 ± 0.1765 ^b^	88.5797 ± 0.5728 ^a^
7d	5.2619 ± 0.2906 ^a^	6.0459 ± 0.1415 ^b^	88.6921 ± 0.4191 ^a^
14d	4.3559 ± 0.5869 ^a^	6.0468 ± 0.1505 ^b^	89.5973 ± 0.5023 ^a^
21d	4.9284 ± 0.7086 ^a^	5.5566 ± 0.1346 ^c^	89.5150 ± 0.8431 ^a^
1:2			
0d	7.3689 ± 0.7700 ^a^	9.3160 ± 0.3006 ^a^	83.3150 ± 0.5506 ^d^
1d	5.6508 ± 0.5872 ^a^	8.8685 ± 0.2216 ^ab^	85.4807 ± 0.4424 ^cd^
3d	5.2138 ± 0.9606 ^a^	8.4077 ± 0.0228 ^b^	86.3785 ± 0.9802 ^bc^
7d	4.3722 ± 0.9514 ^a^	7.5922 ± 0.0680 ^c^	88.0355 ± 0.8955 ^abc^
14d	4.8632 ± 0.7073 ^a^	6.3388 ± 0.3690 ^d^	88.7981 ± 0.5444 ^ab^
21d	5.5151 ± 2.1314 ^a^	5.4532 ± 0.4364 ^e^	89.0317 ± 1.6958 ^a^
1:1			
0d	22.3288 ± 0.4770 ^a^	77.6712 ± 0.4770 ^b^	——
1d	17.0742 ± 0.5061 ^b^	82.9258 ± 0.5061 ^a^	——
3d	15.1173 ± 0.7298 ^b^	84.8827 ± 0.7298 ^a^	——
7d	14.7081 ± 0.8399 ^b^	85.2919 ± 0.8399 ^a^	——
14d	14.4424 ± 1.2396 ^b^	85.5576 ± 1.2396 ^a^	——
21d	14.5642 ± 1.7666 ^b^	85.4358 ± 1.7666 ^a^	——

Note: Data in the table were expressed as mean ± standard deviation, and values in the same column with the same letters are not significantly different (*p* < 0.05).

## Data Availability

The original contributions presented in this study are included in the article. Further inquiries can be directed to the corresponding author.
